# Association of long noncoding RNA GAS5 gene polymorphism with progression of diabetic kidney disease

**DOI:** 10.7150/ijms.99545

**Published:** 2024-08-13

**Authors:** Po-Jen Yang, Ke-Hsin Ting, Po-Yu Tsai, Shih-Chi Su, Shun-Fa Yang

**Affiliations:** 1 School of Medicine, Chung Shan Medical University, Taichung, Taiwan.; 2 Department of Family and Community Medicine, Chung Shan Medical University Hospital, Taichung, Taiwan.; 3 Division of Cardiology, Department of Internal Medicine, Changhua Christian Hospital, Yunlin Branch, Yunlin, Taiwan.; 4 Department of Medicine and Nursing, Hungkuang University, Taichung, Taiwan.; 5 Department of Post-Baccalaureate Medicine, College of Medicine, National Chung Hsing University, Taichung, Taiwan.; 6 Institute of Medicine, Chung Shan Medical University, Taichung, Taiwan.; 7 Division of Nephrology, Department of Internal Medicine, Chung Shan Medical University Hospital, Taichung, Taiwan.; 8 Whole-Genome Research Core Laboratory of Human Diseases, Chang Gung Memorial Hospital, Keelung, Taiwan.; 9 Department of Medical Biotechnology and Laboratory Science, College of Medicine, Chang Gung University, Taoyuan, Taiwan.; 10 Department of Medical Research, Chung Shan Medical University Hospital, Taichung, Taiwan.

**Keywords:** growth arrest-specific 5, gene polymorphism, diabetic kidney disease, long non-coding RNA

## Abstract

Diabetic kidney disease (DKD) is a common microvascular complication of diabetes, whose complex etiology involves a genetic component. *Growth arrest-specific 5* (GAS5), a long noncoding RNA (lncRNA) gene, has been recently shown to regulate renal fibrosis. Here, we aimed to explore the potential role of *GAS5* gene polymorphisms in the predisposition to DKD. One single-nucleotide (rs55829688) and one insertion/deletion polymorphism (rs145204276) of *GAS5* gene were surveyed in 778 DKD cases and 788 DKD-free diabetic controls. We demonstrated that diabetic subjects who are heterozygous at rs55829688 (TC; AOR, 1.737; 95% CI, 1.028-2.937; p=0.039) are more susceptible to advanced DKD but not early-staged DKD, as compared to diabetic subjects who are homozygous for the major allele of rs55829688 (TT). Carriers of at least one minor allele (C) of rs55829688 (TC and CC; AOR, 1.317; 95% CI, 1.023-1.696; p=0.033) more frequently suffer from advanced DKD than do those homozygotes for the major allele (TT). Furthermore, in comparison to those who do not carry the minor allele of rs55829688 (TT), advanced DKD patients possessing at least one minor allele of rs55829688 (TC and CC) exhibited a lower glomerular filtration rate, revealing an impact of rs55829688 on renal co-morbidities of diabetes. In conclusion, our data indicate an association of *GAS5* gene polymorphisms with the progression of DKD.

## Introduction

Chronic kidney disease (CKD) and its progression into end-stage renal disease (ESRD) have been associated with premature mortality and recognized as a global healthcare priority 1. As the renal tissue represents one of the key targets of microvascular damage in diabetes, diabetic kidney disease (DKD), a common complication of diabetes that develops in approximately half of cases with type 2 diabetes mellitus (T2DM) and one-third of those with type 1 diabetes mellitus (T1DM) 2, was thought to be caused by a series of metabolic, hemodynamic, and immunological dysfunctions 3. These dysregulated reactions, involving excessive excretion of metabolites due to aberrant glucose catabolism 4, disturbance of the renin-angiotensin-aldosterone system (RAAS) 5, and activation of numerous signaling cascades that are linked to kidney fibrosis 6, 7, oxidative stress 8, 9, complement system 10, and inflammation 11, 12, collectively orchestrate the pathogenesis of DKD, leading to irreversible kidney damage. Distinct risk parameters have been recognized as contributors to DKD. In addition to several non-modifiable risks (such as age, gender, and genetic inheritance), some of these factors, including hyperglycemia, obesity, hypertension, and dyslipidemia, appear possibly modifiable through intensive diabetes care 13. Such complexity of DKD etiology augments the heterogeneity of the disease epidemiology and treatment, thus prompting us for the discovery of novel biomarkers or manipulable pathogenic factors to improve DKD diagnosis and management.

Numerous investigations have revealed a clear genetic component to both diabetes and its co-morbidities 14. In addition, diabetic subjects with a family history of hypertension or cardiovascular disease tend to develop DKD more frequently 15, 16, supporting the notion that genetic parameters, to some extent, confer the predisposition of DKD in T2DM patients. To date, several hundreds of genetic variants associated with T2DM and DKD have been identified from recent large-scale, multi-ancestry studies 17-21. These genes demonstrate the genetic architecture of T2DM and provide pathogenic insights into DKD, particularly in the development of diabetes, albuminuria, and reduced kidney function in different ethnic groups 22. Nevertheless, the spectrum of DKD susceptibility loci is highly heterogeneous and accounts for only a certain proportion of why some subjects develop CKD and some do not 23. Therefore, identification of novel inherited factors relevant to the development and progression of DKD not only facilitates the understanding of the molecular mechanisms of DKD, but also offers reliable molecular targets for early diagnosis and effective treatment.

The *growth arrest-specific 5* (GAS5) gene encodes a long noncoding RNA (lncRNA) that was originally found to regulate cell growth, differentiation, and development 24, 25. In addition to cell growth arrest, *GAS5* promotes cell apoptosis through acting as a decoy to repress activities of the glucocorticoid receptor, which is a transcription factor for inducing the expression of its target genes in diverse glucocorticoid-mediated responses, such as cell growth/survival and energy expenditure 26. As being downregulated in a verity of malignancies, a tumor-suppressive role of *GAS5* has been recognized 27, 28. However, not only implicated in cancer development, a functional association of *GAS5* with renal fibrosis, a pathological feature of CKD characterized by an excessive accumulation and deposition of extracellular matrix (ECM) components 29, was also proposed. Recently, in an animal model of diabetes, *GAS5* was shown to attenuate renal interstitial fibrosis and kidney inflammation by downregulating matrix metalloproteinase-9, a key regulator of ECM remodeling 30. Through sponging specific microRNAs, such suppressive effect of *GAS5* on renal fibrosis was consistently observed 31. These findings suggest a connection between *GAS5* and renal traits in diabetic individuals. Moreover, the single-nucleotide polymorphisms (SNPs) of *GAS5* were reported to be associated with risk of various cancers 32-35. To date, the effect of *GAS5* gene polymorphisms on the risk and progression of DKD remains unexplored, while a genetic association of *GAS5* with two common co-morbidities of diabetes, retinopathy and coronary artery disease, has been detected 36, 37. Here, we aimed to explore the impact of *GAS5* gene variants on the development and progression of DKD.

## Materials and Methods

### Subject enrollment

To explore the influence of *GAS5* gene polymorphisms on the risk of DKD, 778 patients with DKD were recruited in Chung Shan Medical University Hospital, Taichung, Taiwan, with the approval by the institutional review board (CSMUH No: CS2-22145). CKD was defined as either the presence of proteinuria or an estimated glomerular filtration rate (eGFR, determined by using simplified Modification of Diet in Renal Disease equation) of less than 60 mL/min/1.73 m2 in two separate visits 38. For investigating the disease progression, DKD patients were grouped into early (n=689, CKD stage 1-3; with an eGFR ≥ 30) and advanced DKD (n=89, CKD stage 4-5; with an eGFR < 30) based on the level of renal function decline. In addition, 788 diabetic subjects with normal kidney function were enrolled for comparisons. Informed written consent was obtained from each individual participated in this study. Medical and demographic data concerning age, sex, diabetic status, hyperlipidemic condition, and kidney function were collected from each participant.

### Genotyping

Two *GAS5* gene variants, rs55829688 (T/C, promoter region) and rs145204276 (Ins/Del, promoter region), were surveyed based on their potential link with the risk of diverse diseases 36, 39-41. Extraction of genomic DNA from the whole blood was carried out by using QIAamp DNA Blood Mini kit (Qiagen, Valencia, CA, USA). Allelic discrimination of these two *GAS5* variants including rs55829688 (assay ID: C_88335251_10), and rs145204276 (assay ID: C_166593916_10) was evaluated through the TaqMan assay with an ABI StepOne™ Real-Time PCR System (Applied Biosystems, Foster City, CA, USA). Genotyping data were then processed by SDS version 3.0 software (Applied Biosystems).

### Statistical analysis

Comparisons of clinical or demographic information between DKD patients and non-DKD controls were performed by using the Mann-Whitney U test. Interactions of *GAS5* genotypic frequencies with the development and progression of DKD were assessed by multiple logistic regression models after the adjustment for tentative confounding factors. Differences in GFR between groups and *GAS5* expression data from the Genotype-Tissue Expression (GTEx) database 42 were determined with *student* t-test and one-way ANOVA, respectively. A p value of <0.05 was considered statistically significant.

## Results

### Subject characteristics

To explore the influence of *GAS5* gene polymorphisms on the development of DKD, 778 DKD patients and 788 DKD-free diabetic controls were enrolled. Their demographic and clinical features were assessed (**Table 1**). The average age of DKD cases was higher than that of the controls, as no significant difference in gender was found between two groups. In addition to typical signs of renal impairment (reduced GFR, elevated levels of urinal microalbumin and serum creatinine, and increased UACR), the duration of diabetes and levels of hyperglycemia (elevation of HbA1c levels) were higher in DKD patients, as compared to the control group. Moreover, we observed that DKD group exhibited a higher systolic blood pressure and blood triglycerides level in comparison with diabetic subjects with normal kidney function.

### Association of GAS5 gene variants with advanced DKD

To examine the potential interaction between *GAS5* gene polymorphisms and DKD risks, one single-nucleotide (rs55829688) and one insertion/deletion polymorphism (rs145204276) of *GAS5* gene were genotyped in this survey. Genotypic frequencies of each variant between DKD cases and DKD-free diabetic controls were determined. We did not detect any significant association of these two variants with the development of DKD from our study cohorts (**Table 2**). Subsequently, we further conducted stratification analyses based on the severity of renal impairment. We found that diabetic subjects who are heterozygous at rs55829688 (TC; AOR, 1.737; 95% CI, 1.028-2.937; p=0.039) are more likely to develop advanced DKD (**Table 3**), as compared to diabetic subjects who are homozygous for the major allele of rs55829688 (TT). Carriers of at least one minor allele (C) of rs55829688 (TC and CC; AOR, 1.317; 95% CI, 1.023-1.696; p=0.033) more frequently suffer from the advanced form of DKD than do those homozygotes for the major allele (TT). However, we failed to observe any association of rs55829688 with early DKD (**Table 4**). These results implicate a genotypic influence of *GAS5* rs55829688 on promoting the progression of DKD.

### Effect of GAS5 rs55829688 genotypes on GFR across DKD subgroups and GAS5 expression

**Figure 1:**
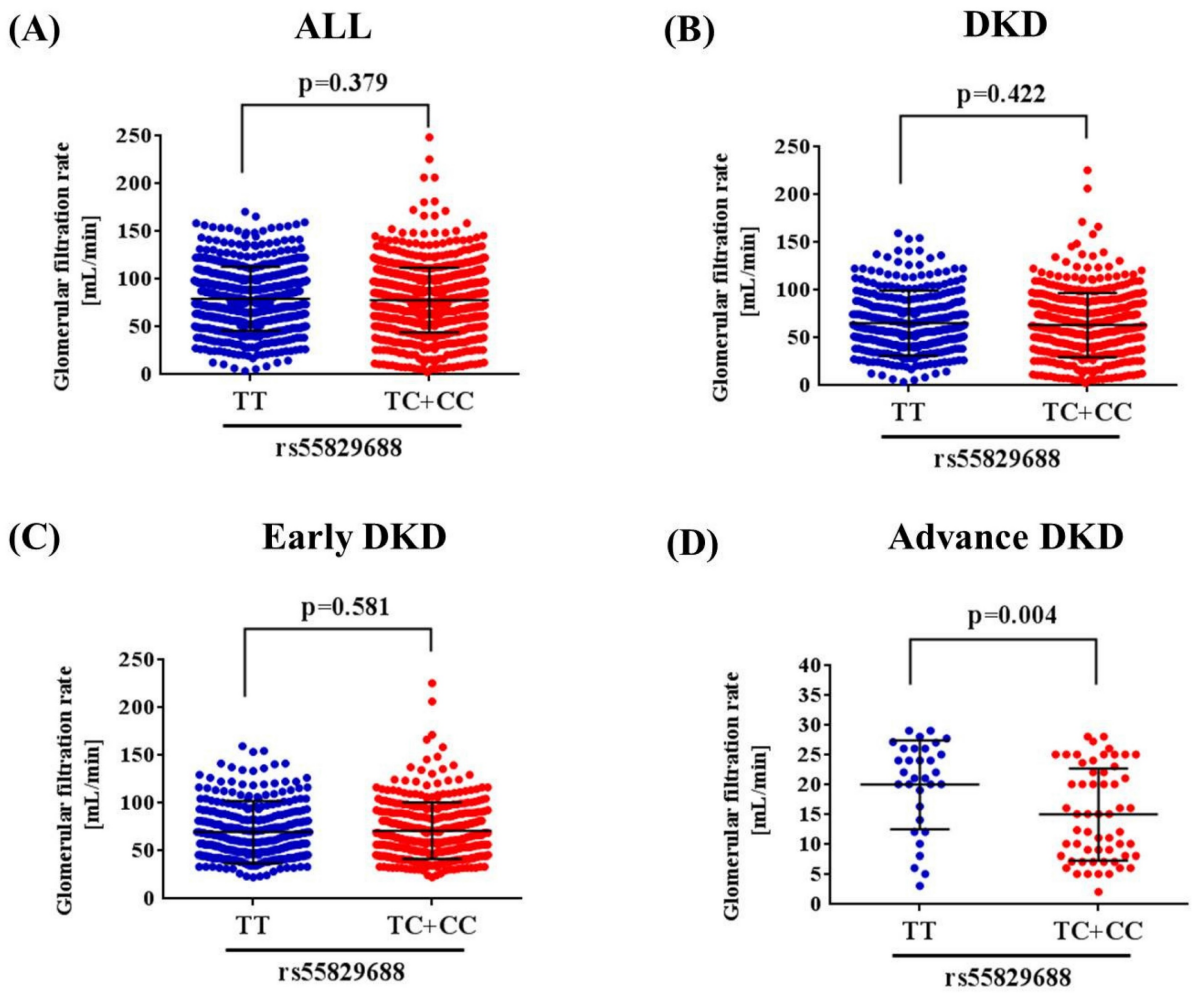
** Effect of rs55829688 genotypes on glomerular filtration rate (GFR) across DKD groups.** Comparisons of GFR between two rs55829688 genotypic groups of all diabetic patients (DKD cases and DKD-free diabetic controls) **(A)**, DKD patients **(B)**, early DKD patients **(C)**, advanced DKD patients **(D)**.

**Figure 2:**
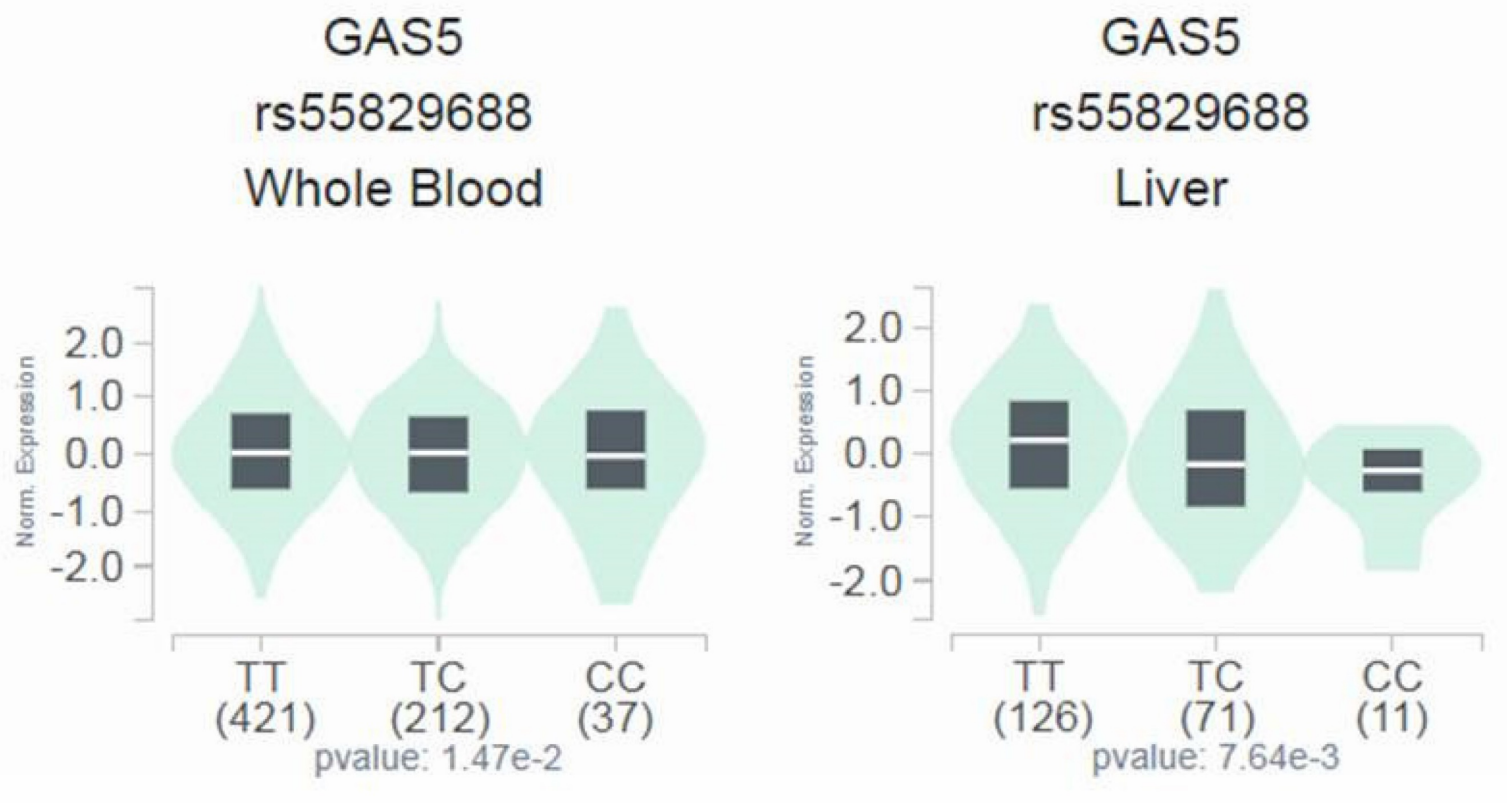
**Effect of rs55829688 genotypes on GAS5 expression.** Comparisons of GAS5 expression among rs55829688 genotypic groups in representative normal tissues based on data from the GTEx portal.

Since a genetic link between* GAS5* rs55829688 and advanced DKD was noted, we next tested whether distinct rs55829688 genotypes affect kidney function of diabetic patients with different levels of renal impairment. We found that the GFR of advanced DKD patients who are homozygous for the major allele of rs55829688 (TT) was significantly higher than that of those who possess at least one minor allele (C) of rs55829688 (TC and CC) (**Figure 1**). Yet, no difference in GFR was seen between two genotypic groups of early DKD, all DKD, or all diabetic patients (DKD cases and DKD-free diabetic controls), suggesting an impact of rs55829688 on loss of kidney function in patients with severe renal failure. In addition, to have a preliminary assessment of the functional relevance for rs55829688, a publicly available dataset was used to evaluate the relationship between rs55829688 genotypes and *GAS5* expression. We found alterations of *GAS5* expression in the whole blood cells and liver tissues and among individuals who carry different rs55829688 genotypes in the Genotype-Tissue Expression (GTEx) database (**Figure 2**). There data suggest that changes in *GAS5* expression due to genetic polymorphisms may affect the disease progression of DKD.

## Discussion

Tremendous amounts of studies have indicated that the risk of DKD is modulated by the combination of inherited and acquired etiologic factors. In this investigation, by employing a candidate gene strategy, we exhibited a correlation between genotypes of *GAS5* rs55829688 and the risk of developing advanced DKD. Moreover, in patients with severe renal impairment, carriers of at least one minor allele of rs55829688 (TC and CC) showed a lower glomerular filtration rate than those homozygotes for the major allele (TT), unveiling an effect of rs55829688 on renal co-morbidities of diabetes. These findings demonstrate a connection of *GAS5* gene variations with the progression of DKD.

Recently, dysregulation of lncRNAs has been attracting increasing attentions on the development of DKD 43. These DKD-associated lncRNAs regulate inflammation, programmed cell death, and epithelial-mesenchymal transition in key resident cells of the kidney, such as mesangial cells, renal endothelial cells, podocytes, and tubular epithelial cells, serving as potential therapeutic targets of DKD 44. One of such lncRNA genes, *GAS5*, encompassing 12 exons and generating two mature RNA transcripts 24, 45, has been proposed as a tumor-suppressor gene 27 and a key regulator of bone diseases 46. Currently, aberrant expression and function of *GAS5* were extensively studied in the field of renal physiology and pathology. In renal tubular cells, *GAS5* hampered the inflammation, oxidative stress, and pyroptosis induced by the treatment of high glucose 47. In addition to the nephroprotective role in renal inflammation and cell death, *GAS5* interfered with the expression of ECM proteins, collagen type I and fibronectin, to alleviate TGFβ-induced renal fibrosis 48. Through diverse molecular mechanisms (e.g. sponging specific microRNAs, suppressing ECM enzymes, and regulating fibrogenic gene transcription), this inhibitory effect of *GAS5* on renal fibrosis was consistently detected in various studies 30, 31, 49-51. Moreover, knockdown of *GAS5* affected the remodeling of renal arteries via altered communications between endothelial cells and vascular smooth muscle cells 52. Collectively, these findings underline a functional relevance of *GAS5* in the pathogenesis of DKD through an epigenetic regulation of nutrient metabolism, renal inflammation, and angiogenic responses.

In this case-control study, we identified a significant correlation of advanced DKD with a single-nucleotide polymorphism (SNP) of *GAS5* gene, rs55829688. This SNP, located at the promoter region of the *GAS5* gene, has been shown to affect the prognosis of acute myeloid leukemia (AML) 53, as specific haplotypes containing rs55829688 were linked to a higher risk of developing AML 54. In addition to the risk and treatment outcome of AML, rs55829688 variation has been demonstrated to confer the susceptibility to colorectal carcinoma 41. Recently, a genetic effect of rs55829688 on the treatment responses of patients with coronary artery disease, another common co-morbidities of diabetes associated with microvascular dysfunction, has also been detected 55. Functional investigations of rs55829688 reveal that genotypes of this variant were able to regulate the expression levels of *GAS5* in peripheral blood and colon cancer cells through altered binding affinities of *GAS5* promoter with the transcription factor p63 53 and Yin Yang-1 41, respectively. Such changes in *GAS5* expression simultaneously manipulated the transcriptional process of its target genes via acting as a sponge for numerous microRNAs and as a scaffold for the formation of multiple transcription factor complexes, eventually resulting in various human disorders 28, 46. Our findings, together with the results from others, suggest that alterations of *GAS5* levels owing to polymorphic alleles of rs55829688 may influence the progression of DKD.

Here, we demonstrated a connection of* GAS5* gene variations with the progression of DKD. Yet, additional efforts are required to deal with several study limitations. One concern is that the highly heterogeneous complications of diabetes (e.g. diabetic retinopathy, diabetic neuropathy, diabetic cardiomyopathy, and diabetic myopathy) and their overlaying genetic architectures may lead to different discoveries regarding the association of *GAS5* gene polymorphisms with advanced DKD. Nevertheless, disease-associated variants within the promoter region were commonly reported as expression quantitative trait loci 56 but we did not examine whether polymorphic alleles of rs55829688 contribute to altered *GAS5* expression in relevant cell types, such as tubular epithelial cells, podocytes, mesangial cells, and renal endothelial cells. In addition, the genetic effect identified in our study might be restricted to specific cohorts if not replicated in other ethnic groups.

Taken together, our data revealed a correlation of *GAS5* rs55829688 with the severe form of DKD. This genetic association links fluctuations of *GAS5* expression owing to gene variations to the exacerbation of renal failure in diabetic individuals.

## Figures and Tables

**Table 1:** Clinical and laboratory characteristics of patients with diabetic kidney disease in diabetic patients.

Variable	No diabetic kidney disease (N=788)	Diabetic kidney disease (N=778)	p value
Age (years)	58.80 ± 11.91	64.10 ± 11.66	<0.001
Male gender [n (%)]	427 (54.2%)	419 (53.9%)	0.895
Duration of diabetes (years)	8.29 ± 6.65	12.09 ± 8.32	<0.001
HbA1c [% (mmol/mol)]	6.96 ± 1.17	7.51 ± 1.51	<0.001
Body mass index [kg/m^2^]	25.92 ± 4.43	26.26 ± 4.54	0.131
Systolic blood pressure [mmHg]	130.86 ± 14.52	137.11 ± 17.23	<0.001
Diastolic blood pressure [mm Hg]	76.30 ± 10.62	76.11 ± 11.76	0.740
Serum creatinine [mg/dL]	0.81 ± 0.29	1.44 ± 1.46	<0.001
Glomerular filtration rate [ml/min]	92.44 ± 26.78	63.76 ± 33.78	<0.001
Total cholesterol [mmol/L]	161.29 ± 40.33	161.65 ± 48.09	0.873
HDL cholesterol [μmol/L]	46.79 ± 12.81	44.12 ± 12.85	<0.001
LDL cholesterol [μmol/L]	87.50 ± 30.43	84.21 ± 31.43	0.037
Triglycerides, [μmol/L]	133.26 ± 185.12	157.54 ± 161.25	0.006
TC/HDL ratio	3.65 ± 1.39	3.94 ± 2.14	0.002
Microalbumin (mg/dL)	1.16 ± 1.24	45.86 ± 98.98	<0.001
UACR (mg/g)	9.90 ± 7.10	551.87 ± 1291.52	<0.001

**Table 2:** Association between GAS5 genotypic frequencies and diabetic kidney disease.

Variable	No diabetic kidney disease (N=788)	diabetic kidney disease (N=778)	AOR (95% CI)	p value
**rs55829688**				
TT	400 (50.8%)	362 (46.5%)	1.000 (reference)	
TC	317 (40.2%)	332 (42.7%)	1.345 (0.841-2.153)	p=0.216
CC	71 (9.0%)	84 (10.8%)	1.503 (0.699-3.233)	p=0.297
TC+CC	388 (49.2%)	416 (53.5%)	1.172 (0.938-1.465)	p=0.163
**rs145204276**				
Ins/Ins	327 (41.5%)	324 (41.6%)	1.000 (reference)	
Ins/Del	362 (45.9%)	371 (47.7%)	1.020 (0.631-1.648)	p=0.936
Del/Del	99 (12.6%)	83 (10.7%)	0.677 (0.328-1.395)	p=0.290
Ins/Del + Del/Del	461 (58.5%)	454 (58.4%)	0.965 (0.769-1.210)	p=0.755

The adjusted odds ratio (AOR) with their 95% confidence intervals were estimated by multiple logistic regression models.

**Table 3:** Association of advanced diabetic kidney disease with GAS5 genotypic frequencies.

Variable	No diabetic kidney disease (N=788)	Advanced diabetic kidney disease (N=89)	AOR (95% CI)	p value
**rs55829688**				
TT	400 (50.8%)	32 (36.0%)	1.000 (reference)	
TC	317 (40.2%)	47 (52.8%)	1.737 (1.028-2.937)	p=0.039
CC	71 (9.0%)	10 (11.2%)	1.727 (0.717-4.159)	p=0.223
TC+CC	388 (49.2%)	57 (64.0%)	1.317 (1.023-1.696)	p=0.033
**rs145204276**				
Ins/Ins	327 (41.5%)	40 (44.9%)	1.000 (reference)	
Ins/Del	362 (45.9%)	42 (47.2%)	0.871 (0.519-1.462)	p=0.602
Del/Del	99 (12.6%)	7 (7.9%)	0.558 (0.224-1.389)	p=0.210
Ins/Del + Del/Del	461 (58.5%)	49 (55.1%)	0.898 (0.700-1.152)	p=0.397

The adjusted odds ratio (AOR) with their 95% confidence intervals were estimated by multiple logistic regression models.

**Table 4:** Association of early diabetic kidney disease with GAS5 genotypic frequencies.

Variable	No diabetic kidney disease (N=788)	Early diabetic kidney disease (N=689)	AOR (95% CI)	p value
**rs55829688**				
TT	400 (50.8%)	330 (47.9%)	1.000 (reference)	
TC	317 (40.2%)	285 (41.4%)	1.347 (0.842-2.156)	p=0.215
CC	71 (9.0%)	74 (10.7%)	1.505 (0.700-3.237)	p=0.295
TC+CC	388 (49.2%)	359 (52.1%)	1.173 (0.938-1.466)	p=0.161
**rs145204276**				
Ins/Ins	327 (41.5%)	284 (41.2%)	1.000 (reference)	
Ins/Del	362 (45.9%)	329 (47.8%)	1.021 (0.632-1.649)	p=0.933
Del/Del	99 (12.6%)	76 (11.0%)	0.677 (0.328-1.395)	p=0.290
Ins/Del + Del/Del	461 (58.5%)	405 (58.8%)	0.965 (0.769-1.210)	p=0.757

The adjusted odds ratio (AOR) with their 95% confidence intervals were estimated by multiple logistic regression models.
